# Generation and characterization of two immortalized dermal fibroblast cell lines from the spiny mouse (*Acomys*)

**DOI:** 10.1371/journal.pone.0280169

**Published:** 2023-07-07

**Authors:** Michele N. Dill, Mohammad Tabatabaei, Manasi Kamat, Kari B. Basso, Erika Moore, Chelsey S. Simmons

**Affiliations:** 1 J. Crayton Pruitt Family Department of Biomedical Engineering, University of Florida, Gainesville, Florida, United States of America; 2 Department of Mechanical and Aerospace Engineering, University of Florida, Gainesville, Florida, United States of America; 3 Department of Chemistry, University of Florida, Gainesville, Florida, United States of America; 4 Department of Materials Science and Engineering, University of Florida, Gainesville, Florida, United States of America; University of Minnesota Medical School, UNITED STATES

## Abstract

The spiny mouse (*Acomys*) is gaining popularity as a research organism due to its phenomenal regenerative capabilities. *Acomys* recovers from injuries to several organs without fibrosis. For example, *Acomys* heals full thickness skin injuries with rapid re-epithelialization of the wound and regeneration of hair follicles, sebaceous glands, erector pili muscles, adipocytes, and dermis without scarring. Understanding mechanisms of *Acomys* regeneration may uncover potential therapeutics for wound healing in humans. However, access to *Acomys* colonies is limited and primary fibroblasts can only be maintained in culture for a limited time. To address these obstacles, we generated immortalized *Acomys* dermal fibroblast cell lines using two methods: transfection with the SV40 large T antigen and spontaneous immortalization. The two cell lines (AcoSV40 and AcoSI-1) maintained the morphological and functional characteristics of primary *Acomys* fibroblasts, including maintenance of key fibroblast markers and ECM deposition. The availability of these cells will lower the barrier to working with *Acomys* as a model research organism, increasing the pace at which new discoveries to promote regeneration in humans can be made.

## Introduction

The spiny mouse (*Acomys*) is gaining popularity as a research organism, largely due to its phenomenal regenerative capabilities [1]. *Acomys* has been shown to regenerate damage to the skin [2–7], ear pinna [8–10], skeletal muscle [11], kidney [12], heart [13–15], and spinal cord [16,17]. Remarkably, all these findings have occurred within the last decade. The increased interest of *Acomys* as a research organism and its potential for future regenerative medicine applications creates a need for research tools that can be used to increase the accessibility of *Acomys* as a model organism and promote collaboration among researchers across different fields and universities.

However, the use of emerging research organisms has challenges. Many researchers do not have access to non-traditional animal facilities, and there are higher associated costs with maintaining non-traditional animal colonies. In the case of *Acomys*, the obstacles are even greater, as they are not sold by commonly used vendors like the Jackson laboratory and their colonies require maintenance procedures that differ from those of commonly housed rodents [1,18–20]. In addition, *Acomys* have longer gestation periods and smaller litters which makes building and maintaining a colony difficult. The limited presence and size of *Acomys* colonies available for research limits access to *Acomys* even as interest continues to increase.

Using primary cell cultures from *Acomys* carries similar challenges as primary cells require access to an *Acomys* colony, and repeated cell isolation can place stress on colonies being used for multiple projects. In addition, primary and secondary cell cultures can only be maintained *in vitro* for a short period of time before they enter replicative senescence and cell division is no longer possible [21,22]. Even prior to replicative senescence, primary and secondary cell cultures begin to show morphological and functional changes which limits their use to early passages. Isolation of primary cells is also time consuming, and the initial population of cells may be heterogeneous. While sorting for the cell type of interest is typically an option when working with primary isolates, the poor cross-reactivity of many antibodies presents even more challenges in *Acomys* [1,23,24].

In comparison to primary and secondary cell cultures, immortal cell lines have ‘infinite’ culturing capabilities and off-the-shelf availability, meaning they can easily be shared across research institutions. Immortal cell lines have acquired the ability to proliferate indefinitely through either artificial genetic modifications or spontaneous mutations. They are widely used because they are easy and inexpensive to maintain, manipulate, and expand. Immortal cells are a valuable tool for preliminary experiments because they are a pure population of cells which improves reproducibility. In addition, they obviate the need for consistent isolation of primary cells to support *in vitro* experiments.

Cell immortalization through artificial genetic modifications is often performed by transfection with viral oncogenes. Immortalization of cells through transfection with the simian virus 40 large T antigen (SV40-LT) has been used for decades and is described as a simple and reliable agent for the immortalization of many different cell types, and there are over 170 SV40-LT transfected cell lines available through ATCC alone [25–28]. Although the SV40-LT binds to many proteins, its interactions with the tumor suppressors retinoblastoma (Rb) and p53 are essential for bypassing replicative senescence [25,29]. Rb and p53 serve to prevent excessive cell growth and mutations by halting cell cycle progression, and their inhibition by SV40-LT promotes cell immortalization by preventing senescence. Cells transfected with SV40-LT also demonstrate stabilization of telomere length due to increased telomerase activity [30]. Viral transfection is not complete in all cells. Instead, within a cell culture, there are nonpermissive infections, semipermissive infections, and full transformations [31,32]. Fully transformed cells will eventually overtake the other populations in culture, but they can also be reliably isolated using selectable markers like antibiotic resistance genes, producing a culture of transformed cells.

Cells can also be immortalized through spontaneous mutations that result from prolonged subculture and genomic instability. Spontaneous immortalization of normal human cell lines is rare whereas normal rodent cells can regularly be established spontaneously [33,34]. One example is NIH3T3 fibroblasts which have been utilized in research since they were first developed by Todaro and Green in 1963 [35]. Loss of a key tumor suppressor gene like p53 is necessary for spontaneous immortalization, but another mutation such as chromosomal recombination or epigenetic silencing is also required [36,37]. Interestingly, the mutations required to escape replicative senescence can change with cell type and culture conditions [38–40], making it difficult to point to any specific mutations as being the key to spontaneous immortalization. Nonetheless, a variety of cell lines from different organisms including mice, humans, pigs, and chickens have been derived through spontaneous immortalization and utilized in experimentation [41–44].

To support the regenerative medicine community and to reduce the number of animals used in research, we sought to generate immortalized *Acomys* fibroblasts. We chose to focus on the dermis because regeneration of *Acomys* skin has been widely reported [2–7], but exact mechanisms remain elusive. Strikingly, *Acomys* can regenerate hairs, sebaceous glands, erector pili muscles, adipocytes, and the panniculus carnosus following full thickness excision wounds [2] and burn wounds [5] to the skin. We have chosen to focus on fibroblasts because we hypothesize that fibroblast activation plays a notable role in *Acomys* regeneration [45], and we are investing in *in vitro* experimental platforms to uncover specific regenerative mechanisms. We generated two *Acomys* dermal fibroblast lines—one through transfection with SV40-LT and one through extended subculture—and verified their functional similarity to primary *Acomys* fibroblasts (pAFs). These cell lines have been accepted into the ATCC general collection and licensed by abm.

## Materials and methods

### Isolation of pAFs

Care and use of animals was conducted in accordance with the United States Department of Agriculture (USDA) and National Institutes of Health (NIH) guidelines and were approved by the UF Institutional Animal Care and Use Committee. *Acomys cahirinus* and CD-1 *Mus musculus* pups were obtained from UF breeding colonies.

*Acomys* pups were euthanized within 3 days of birth and the dorsal skin was removed. The tissue was incubated overnight in 0.2% dispase II (Roche) in DMEM at 4°C, after which the dermis and epidermis were separated with forceps. The dermis was dissociated by incubation in 0.24% collagenase type I (Gibco) in PBS at 37°C for 60–90 minutes with constant agitation. A cell suspension was obtained using a vacuum filtered conical tube, and the cells were rinsed and cultured in DMEM/F:12 supplemented with 10% fetal bovine serum (Gibco), 10% NuSerum (Corning), 1% Gentamicin/Amphotericin B (Gibco), and 0.1% insulin-transferrin-selenium (Gibco). Isolation of cells from CD-1 *Mus musculus* pups followed the same procedure.

### SV40LT transfection of pAFs

Fibroblasts were isolated from an *Acomys* neonate and expanded for two passages on soft silicone (Sylgard^TM^ 527 Silicone Dielectric Gel, Dow Inc.) coated with 0.01mg/mL rat tail collagen I (Corning) prior to being frozen and shipped to ALSTEM, Inc for immortalization. At ALSTEM, cells were cultured in provided collagen-coated Sylgard 527 well plates and using the media formulation described above. Sylgard 527 was used to mimic the stiffness of *in vivo* tissue and avoid phenotypic changes in the pAFs during culture. 100,000 cells were infected with a lentivirus encoding the SV40 large T antigen and puromycin N-acetyltransferase. The cells were selected by puromycin at 2ug/mL and passaged for 2–3 passages. The expression of SV40 and puromycin resistance genes was confirmed via PCR (S1 Fig) before the cells were frozen.

Upon receiving the cells, continuous proliferation was confirmed by passaging the cells at a constant interval (3 days) and seeding density (5,300 cells/cm^2^). Growth curves were generated by calculating the population doubling (PDL) at each passage using the formula PDL = PDL_0_ + 3.32(LogCf−LogC_0_) where C_f_ is the final cell count at the time of passaging and C_0_ is the seeding number. The cells were intermittently frozen so proliferation could be assessed across both passages and freeze/thaw cycles. We utilized the media formulation that has been optimized for our pAFs (described above) for both immortalized lines to directly compare them to pAFs. However, we have demonstrated that both lines also retain continuous proliferation in the presence of a more commonly used formulation (S2 Fig).

### Spontaneous immortalization of primary *Acomys* fibroblasts

Fibroblasts were isolated from three *Acomys* neonates from separate litters and cultured until logarithmic growth was re-established following a period of reduced proliferation. Cells were passaged at 80% confluency, and a constant seeding density (5,300 cells/cm^2^) was used. The cells were classified as immortalized once proliferation increased for 3 passages following the period of reduced proliferation, or crisis.

One set of immortalized *Acomys* fibroblasts (AcoSI-1) was chosen for further characterization. Continuous proliferation of AcoSI-1 cells was confirmed by passaging the cells at a constant interval (3 days) and seeding density (5,300 cells/cm^2^). Growth curves were generated by calculating the PDL at each passage as described above. The cells were intermittently frozen so proliferation could be assessed across both passages and freeze/thaw cycles.

### Assessment of fibroblast markers via western blot

Cell lysates were prepared in RIPA buffer, and equal amounts of protein were loaded into NuPage 4–12% Bis-Tris Midi gels (Invitrogen) submerged in NuPage MES SDS Running Buffer (Life Technologies). The proteins were transferred onto a nitrocellulose membrane using the SureLock™ Tandem Midi Blot Module (Life Technologies) and NuPage Transfer Buffer (Life Technologies) containing 10% methanol. The membrane was blocked with 5% powdered milk in tris-buffered saline for 1 hour at room temperature. Membranes were incubated with either a mouse monoclonal anti-alpha smooth muscle actin (Abcam ab7817, 0.341ug/mL) or a rabbit monoclonal anti-vimentin antibody (Abcam ab92547, 1:5000 dilution) with a mouse monoclonal anti-GAPDH loading control (Arigo biolaboratories ARG10112, 1:5000 dilution) overnight at 4°C. Incubation with HRP secondary antibodies (Arigo biolaboratories ARG65350, 1:5000 dilution or enQuire BioReagents QAB10303, 1:15,000 dilution) was performed for 1 hour at room temperature. Signal was produced using SuperSignal^TM^ West Pico PLUS Chemiluminescent Substrate (Thermo Scientific) and the blots were imaged on a LI-COR Fc Imager (Odyssey).

### Assessment of contractility with traction force microscopy

Cell contractility was evaluated by using traction force microscopy (TFM) as described previously [46]. Cells were seeded on 8kPa polyacrylamide hydrogels coated with fluorescent nano-beads (Cell&Soft) and allowed to adhere for 12 hours. The samples were then mounted on a Nikon microscope with a 37°C chamber, and DMEM media was replaced with CO_2_-independent media (Leibovitz) prior to the test. Fluorescent images of the nano-beads were captured before and after cell detachment with a 1% Triton-X and 200 mM KOH solution. Bead displacement was quantified and used to calculate the root-mean-squared values of stress and strain energy. For each group of tests, at least 50 cells were measured and results were reported as the mean ± standard deviation. The results were compared using one-way analysis of variance (ANOVA) with the Bonferroni post hoc test. p‐value < 0.05 was considered statistically significant.

### Preparation of cell derived matrices

Cell derived matrices (CDMs) were obtained by seeding pAFs, AcoSV40, or AcoSI-1 fibroblasts onto collagen-functionalized Sylgard 527 PDMS. Primary *Mus* fibroblasts and NIH3T3 fibroblasts were included as controls, and 3 sets of CDMs were made from each cell type (15 samples total). Prior to cell seeding, the substrate was plasma treated to oxidize the surface before functionalizing with (3-Aminopropyl)trimethoxysilane, followed by glutaraldehyde and then rat tail collagen I. Functionalization with collagen I is needed to prolong adherence of *Acomys* cells to the substrate. Cells were cultured for 7–28 days in media containing 25mg/mL Ficoll 400, with immortalized cells requiring less time in culture to generate similar protein mass as primary cells. Following this period, CDMs were decellularized by treating with a solution of 0.5%Triton X-100 and 0.3M ammonium hydroxide in PBS for 5 minutes. The presence of residual DNA was reduced by treating decellularized CDMs with 10ug/mL DNase I at 37oC for 30 minutes.

Decellularized CDMs were homogenized with RIPA Buffer and a tissue homogenizer (Fisherbrand^TM^ 150 Handheld Homogenizer). The samples were centrifuged at 3200g and 4°C for 15 minutes, followed by 14,000g for 2 minutes. The supernatant was removed and stored at -20°C as the RIPA soluble protein fraction. The pellet was resuspended in a membrane solubilization buffer containing 40mM Tris-Cl (pH 8.0), 7M urea, 2M thiourea, 0.25% w/v ASB-14, and 0.25% NP-40 and incubated at room temperature for 15 minutes. The samples were vortexed for 1 minute, then centrifuged at 3200g for 30 minutes. The supernatant was removed and diluted by 2.5 with dH_2_O and stored at –20°C as the RIPA insoluble protein fraction. Prior to analysis, both the RIPA soluble and RIPA insoluble protein fractions were precipitated in ice-cold acetone overnight. The solutions were centrifuged at 3200g and 4°C for 15 minutes. The acetone was fully removed, and the remaining pellets were resuspended in 2M urea. Equal concentrations of the RIPA soluble and RIPA insoluble protein fractions from each sample were combined, and label-free quantitative proteomics was performed by the UF Mass Spectrometry Research and Education Center.

### Label-free quantitative proteomics of CDM samples

#### In solution digestion

Total protein was determined on a Qubit and the appropriate volume of each sample was taken to equal 20 μg total protein for digestion. The samples were digested with sequencing grade trypsin/lys C enzyme (Promega) using manufacture recommended protocol. The samples were diluted with 50mM ammonium bicarbonate buffer. The samples were incubated at 56°C with 1.0 μL of dithiothreitol (DTT) solution (0.1 M in 50 mM ammonium bicarbonate) for 30 minutes prior to the addition of 3.0 μL of 55 mM iodoacetamide in 50 mM ammonium bicarbonate. Samples with iodoacetamide were incubated at room temperature in the dark for 30 min. The trypsin/lys C was prepared fresh as 1 μg/μL in reconstitution buffer (provided with the enzyme). 1 μL of enzyme was added and the samples were incubated at 37°C overnight. The digestion was stopped with addition of 0.5% trifluoracetic acid. The MS analysis is immediately performed to ensure high quality tryptic peptides with minimal non-specific cleavage.

#### Q Exactive HF Orbitrap

Nano-liquid chromatography tandem mass spectrometry (Nano-LC/MS/MS) was performed on a Thermo Scientific Q Exactive HF Orbitrap mass spectrometer equipped with an EASY Spray nanospray source (Thermo Scientific) operated in positive ion mode. The LC system was an UltiMate™ 3000 RSLCnano system from Thermo Scientific. The mobile phase A was water containing 0.1% formic acid and the mobile phase B was acetonitrile with 0.1% formic acid. The mobile phase A for the loading pump was water containing 0.1% trifluoracetic acid. 5 mL of sample is injected on to a PharmaFluidics mPAC^TM^ C18 trapping column (C18, 5 μm pillar diameter, 10 mm length, 2.5 μm inter-pillar distance). at 25 μl/min flow rate. This was held for 3 minutes and washed with 1% B to desalt and concentrate the peptides. The injector port was switched to inject, and the peptides were eluted off of the trap onto the column. PharmaFluidics 50 cm mPAC^TM^ was used for chromatographic separations (C18, 5 μm pillar diameter, 50 cm length, 2.5 μm inter-pillar distance). The column temperature was maintained 40°C. A flow-rate of 750 nL/min was used for the first 15 minutes and then the flow was reduced to 300 nL/min. Peptides were eluted directly off the column into the Q Exactive system using a gradient of 1% B to 20%B over 100 minutes and then to 45%B in 20 minutes for a total run time of 150 minutes.

The MS/MS was acquired according to standard conditions established in the lab. The EASY Spray source operated with a spray voltage of 1.5 KV and a capillary temperature of 200°C. The scan sequence of the mass spectrometer was based on the original TopTen™ method; the analysis was programmed for a full scan recorded between 375–1575 Da at 60,000 resolution, and a MS/MS scan at resolution 15,000 to generate product ion spectra to determine amino acid sequence in consecutive instrument scans of the fifteen most abundant peaks in the spectrum. The AGC Target ion number was set at 3e6 ions for full scan and 2e5 ions for MS^2^ mode. Maximum ion injection time was set at 50 ms for full scan and 55 ms for MS^2^ mode. Micro scan number was set at 1 for both full scan and MS^2^ scan. The HCD fragmentation energy (N)CE/stepped NCE was set to 28 and an isolation window of 4 *m/z*. Singly charged ions were excluded from MS^2^. Dynamic exclusion was enabled with a repeat count of 1 within 15 seconds and to exclude isotopes. A Siloxane background peak at 445.12003 was used as the internal lock mass.

HeLa protein digest standard is used to evaluate the integrity and the performance of the columns and mass spectrometer. If the number of protein ID’s from the HeLa standard falls below 2700, the instrument is cleaned and new columns are installed.

All MS/MS spectra were analyzed using Sequest (Thermo Fisher Scientific; version IseNode in Proteome Discoverer 2.4.0.305). Sequest was set up to search *Mus saxicola* Tax ID: 10094 assuming the digestion enzyme trypsin. Sequest was searched with a fragment ion mass tolerance of 0.020 Da and a parent ion tolerance of 10.0 ppm. Carbamidomethyl of cysteine was specified in Sequest as a fixed modification. Met-loss of methionine, met-loss+Acetyl of methionine, oxidation of methionine and acetyl of the n-terminus were specified in Sequest as variable modifications.

Precursor ion intensity label free quantitation was done using Proteome Discoverer (Thermo Fisher Scientific vs 2.4.0.305). The two groups (B33p4 vs Hp4) were compared using a “non-nested” study factor. Normalization was derived by using all peptides. Protein abundances were calculated by summed abundances, meaning the protein abundances are calculated by summing sample abundances of the connected peptide groups. Fisher’s exact test (pairwise ratio-based) was used to calculate p-values with no missing value imputation included. Adjusted p-values were calculated using Benjamini-Hochberg.

### Determining sex of immortalized *Acomys* fibroblast lines

Genomic DNA was isolated from 1 million cells of each cell line using the QIAamp^®^ DNA Micro Kit (QIAGEN) following the manufacturer’s guidelines. Control gDNA was obtained from the peripheral blood of an adult male *Acomys*. DNA quantity was determined using a BioTek Synergy H1 plate reader (Agilent Technologies), and PCR reactions were performed using *Acomys*-specific primers for the target SrY. The PCR products were added to 2% E-Gel agarose gels with SYBR Safe DNA Stain (Invitrogen) and run in an E-Gel PowerSnap system (Invitrogen). Images were acquired using a LI-COR Fc Imager (Odyssey).

## Results

### Immortalized *Acomys* fibroblasts demonstrate continuous proliferation

**Fig 1:**
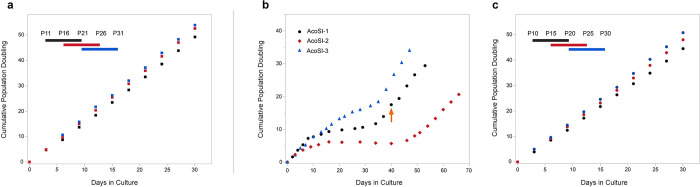
Immortalized AcoSV40 and AcoSI-1 fibroblasts demonstrate logarithmic growth over at least 30 passages. (a) AcoSV40 fibroblasts maintain logarithmic growth across 30 passages and 3 freeze/thaw cycles. (b) pAFs subjected to continuous subculture spontaneously immortalize after about 40 days. (c) AcoSI-1 fibroblasts maintain logarithmic growth across 30 passages and 3 freeze/thaw cycles.

Primary *Acomys* fibroblasts (pAFs) were isolated, cultured on silicone-coated tissue culture plates (Sylgard 527, Elastic Modulus ~5 kPa), and sent to ALSTEM, Inc for immortalization with the SV40 large T antigen via lentiviral infection. The cells were selected by puromycin, and successful immortalization was confirmed through PCR assessment of SV40 and puromycin resistance genes (S1 Fig). We refer to these cells as “AcoSV40” cells. Continuous proliferation was confirmed by passaging the cells at a constant interval (3 days) and seeding density (5,300 cells/cm^2^) and calculating the population-doubling level at each passage. The AcoSV40 cells demonstrated continuous proliferation over 30 passages and multiple freeze/thaw cycles (Fig 1A).

Spontaneous immortalization of pAFs was performed through extended subculturing where the seeding density was kept constant (5,300 cells/cm^2^), and the cells were passaged upon reaching 80–90% confluency. As shown in Fig 1B, the fibroblasts demonstrated consistent proliferation for several passages prior to experiencing a period of ‘crisis’ during which there was a progressive decline in proliferation. A subpopulation of these cells eventually recovered and began to proliferate, overtaking the culture. Once this occurred, the cells were deemed immortalized and were named “AcoSI” cells. pAFs were isolated from three different animals, and the process was repeated to demonstrate replicability. AcoSI cells were generated within 13–17 generations and 40–55 days in culture, similar to reports by Todero and Green, in which mouse endothelial fibroblasts exited crisis after 15–30 generations and 45–75 days in culture [35]. The decreased time to immortalization in *Acomys* fibroblasts may be due in part to higher rates of proliferation in *Acomys* fibroblast cultures when compared to mouse fibroblast cultures (S3 Fig).

AcoSI-1 fibroblasts were chosen for futher assays due to the marked crisis period and median proliferation rates compared to the other two AcoSI lines. The passage at which the *Acomys* fibroblasts were deemed to be immortalized is marked by the arrow in Fig 1B and was recorded as AcoSI-1 passage 1. The same procedure was followed to determine continuous proliferation in the AcoSI-1 cells as the AcoSV40 cells. The cells were passaged every three days and seeded at a constant cell density. Population doubling remained consistent over 30 passages and multiple freeze/thaw cycles (Fig 1C).

#### AcoSV40 and AcoSI fibroblasts retain similar morphological characteristics compared to pAFs

**Fig 2:**
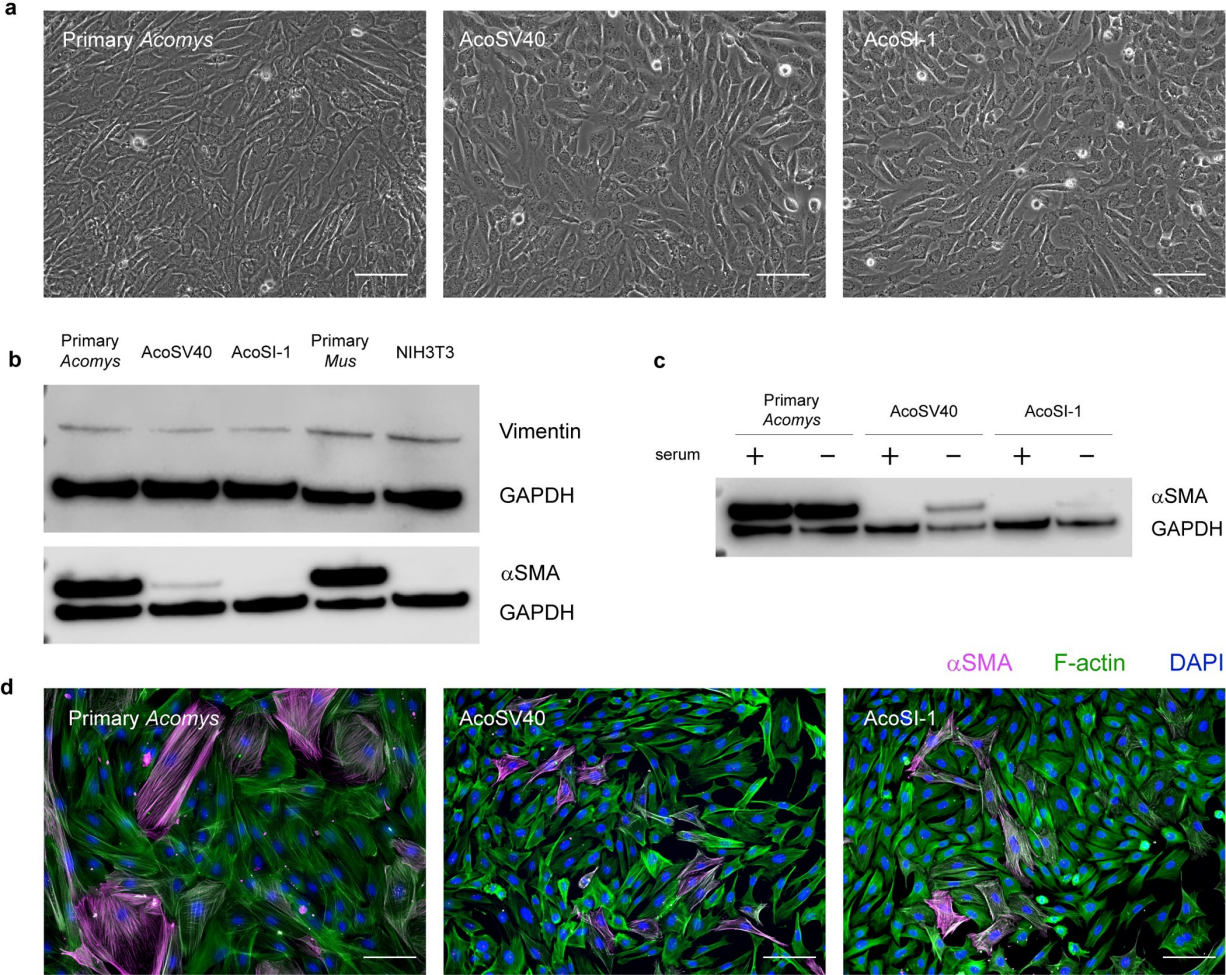
AcoSV40 and AcoSI-1 fibroblasts maintain morphological characteristics and fibroblast markers (vimentin and αSMA) of pAFs. (a) Phase images of passage 1 pAFs, AcoSV40, and AcoSI-1 fibroblast demonstrate similar morphology; scale = 100um. (b) pAFs, AcoSV40, and AcoSI-1 fibroblasts express vimentin at similar levels, but αSMA is weekly present in AcoSV40 and absent in AcoSI-1 fibroblasts under standard culture conditions. We demonstrated that under serum starvation, AcoSV40 and AcoSI-1 can (c) upregulate αSMA and (d) co-localize it to stress fibers; scale = 100μm.

The morphology of the two immortalized *Acomys* fibroblast lines was compared to passage 1 pAFs via phase imaging. As depicted in Fig 2A, all three cell types share a similar size and shape, with the immortal cell lines appearing less spread than the pAFs and more cuboidal in shape.

The AcoSV40 and AcoSI lines were also assessed for the fibroblast markers vimentin and alpha smooth muscle actin (αSMA) to confirm relevant markers are retained during immortalization. Primary *Mus musculus* and immortalized *Mus musculus* (NIH3T3) fibroblasts were included as a comparison. As demonstrated by western blot (Fig 2B), all cell types maintain the vimentin marker and express it at similar levels under normal culture conditions. The AcoSV40 fibroblasts express αSMA at low levels in normal culture conditions compared to pAFs. Interestingly, the AcoSI-1 cells did not produce αSMA bands when assessed via western bot. However, this finding was shared with the NIH3T3 cells, which are also a spontaneously immortalized cell line. NIH3T3 fibroblasts are known to upregulate αSMA in culture when treated with TGF-β1 [47–49], suggesting that AcoSI-1 fibroblasts may also be able to upregulate αSMA under different culture conditions. TGF-β1 was not a good candidate for our purposes due to the lack of response documented in *Acomys* fibroblasts [50], which was confirmed to be maintained in AcoSV40 and AcoSI-1 fibroblasts (S4 Fig). Instead, we assessed αSMA upregulation under serum starvation conditions and found that both AcoSV40 and AcoSI-1 fibroblasts produce more αSMA in the absence of serum, although AcoSI-1 cells make only a small quantity (Fig 2C). Both AcoSV40 and AcoSI-1 cells were also able to co-localize αSMA to stress fibers (Fig 2D), supporting their utility in fibroblast activation studies.

### AcoSV40 and AcoSI-1 fibroblasts retain functional characteristics of pAFs

**Fig 3:**
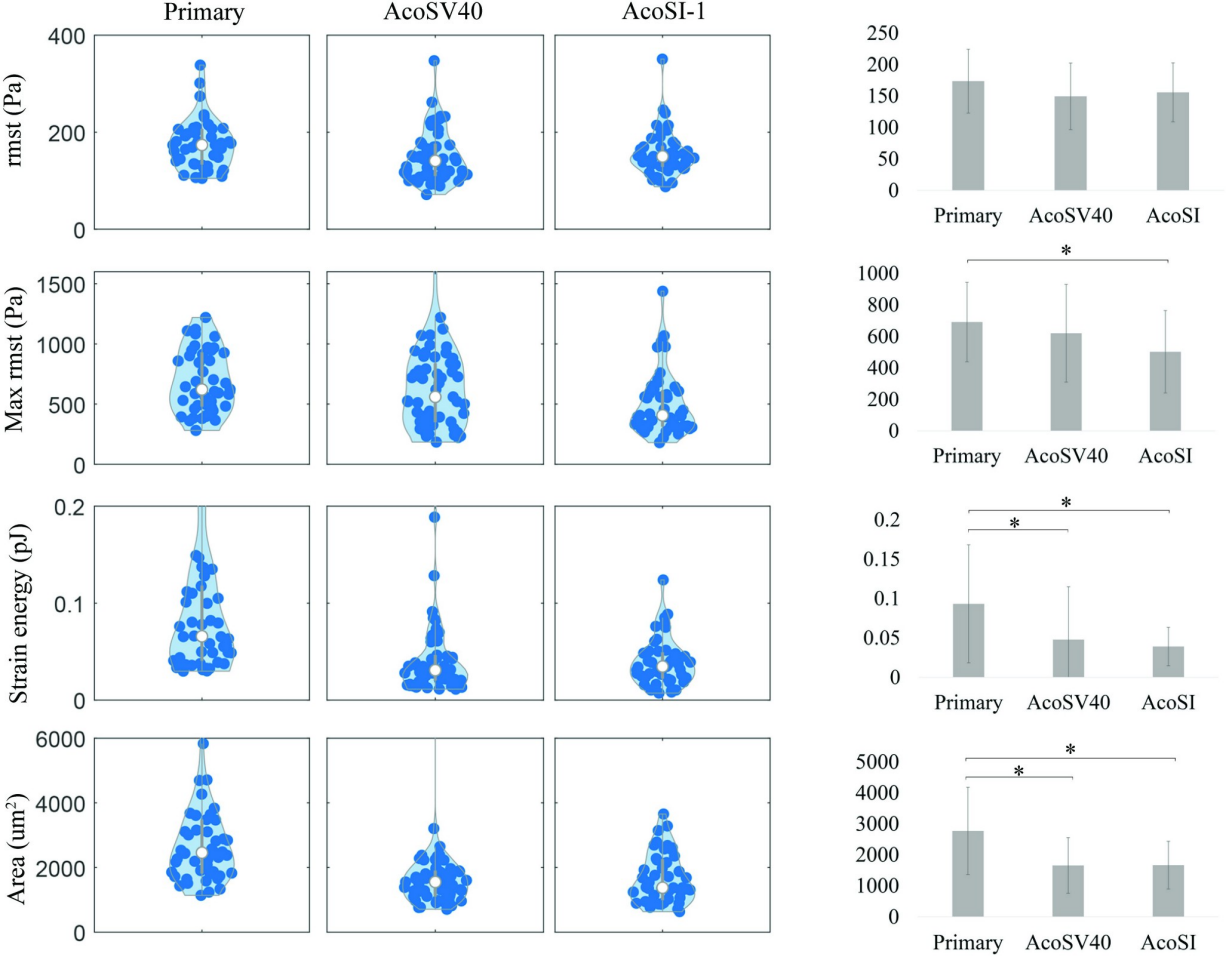
Traction forces of pAFs, AcoSV40, and AcoSI-1 fibroblasts are similar, and differences in strain energy correlate with differences in cell area. Cells were seeded on 8kPa polyacrylamide gels to evaluate root-mean-square of traction (rmst), max rmst, strain energy, and area. pAFs have higher maximum contraction stresses than AcoSI-1 cells, but similar average stresses to both immortalized lines. While pAFs have higher strain energy (i.e., do more work) than the immortalized fibroblasts, it is likely a function of their larger area. *p<0.05 as assessed by ANOVA with Bonferroni test.

Contractility and deposition of extracellular matrix (ECM) are important functional characteristics of fibroblasts and play a key role in stabilization of the wound bed following injury. We assessed the contractility of AcoSV40 and AcoSI-1 fibroblasts through traction force microscopy and found that they generate similar average (root-mean-square traction, rmst) and maximum (Max rmst) traction forces as pAFs (Fig 3). Strain energy (integrated traction force and deformation over the area of the cell) is unsurprisingly lower for immortalized fibroblasts since they have a smaller spread area than primary cells. Traction force microscopy confirms maintenance of contractile function in both AcoSV40 and AcoSI-1 immortalized lines.

**Fig 4:**
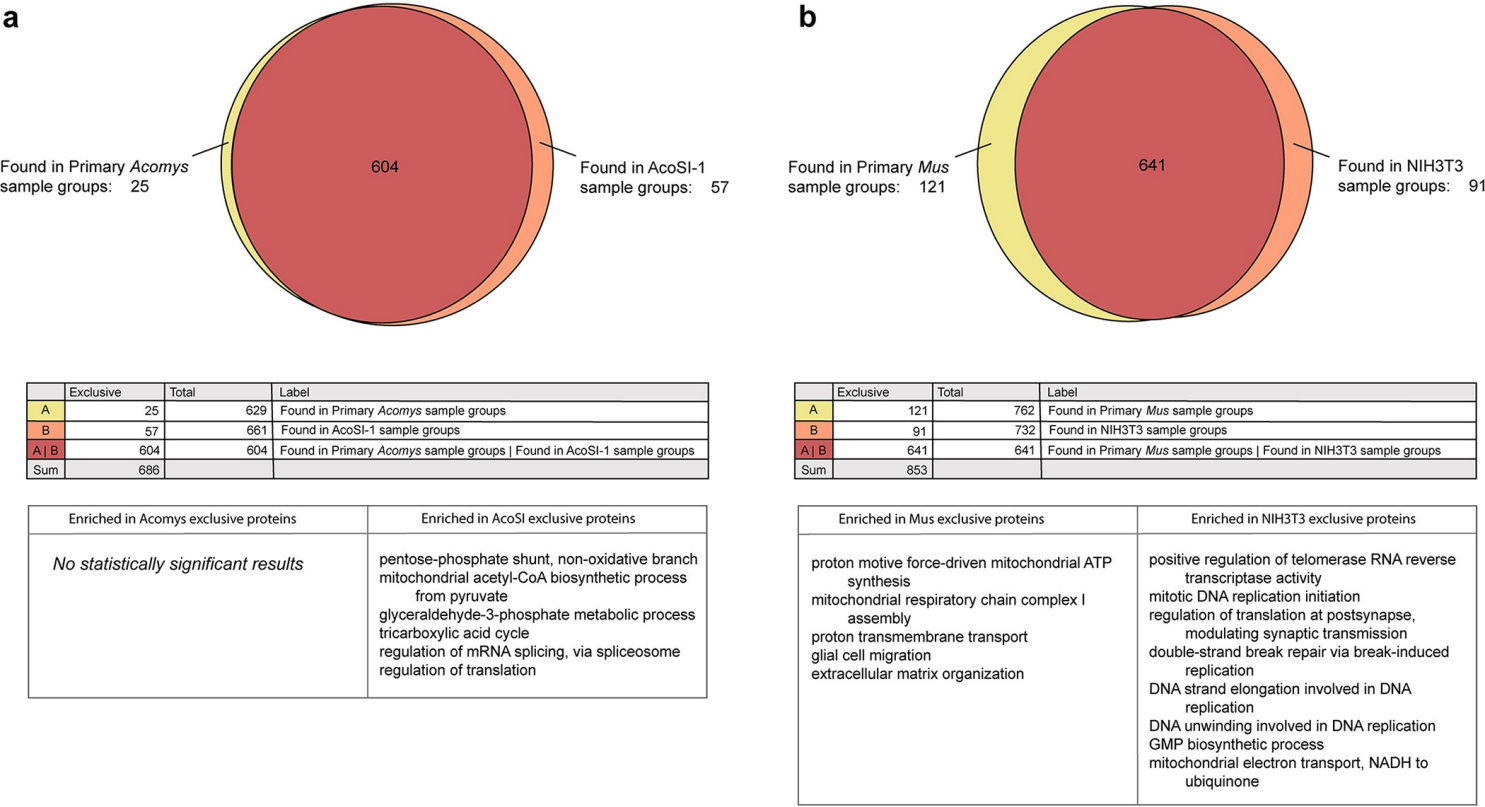
Immortalized lines AcoSV40 and AcoSI-1 share most proteins with pAFs. The comparison of AcoSV40 and pAF CDMs via mass spectrometry resulted in no significant difference in deposited proteins. (a) AcoSI-1 CDMs shared 604 out of 686 proteins identified with pAFs and enriched biological processes within the dissimilar proteins were unrelated to ECM organization. (b) In comparison, NIH3T3 fibroblasts share 641 out of 853 identified proteins with *Mus* primary fibroblasts, some of which are related to ECM organization. GO biological processes were determined by running the proteins that were exclusive to Primary *Acomys*, AcoSI-1, Primary *Mus*, and NIH3T3 fibroblasts through the PANTHER classification system. For brevity, only the top eight enriched biological processes were reported in the NIH3T3 exclusive proteins, all of which had a fold enrichment change of over 50.

Fibroblasts are responsible for maintaining the ECM proteome, composed of hundreds of proteins and proteoglycans which are encoded by over 1000 genes [51]. To capture this complexity, we compared ECM production in primary and immortalized *Acomys* fibroblasts by generating cell derived matrices (CDMs). CDMs recapitulate native tissue structure and composition *in vitro* and are useful for studying bulk matrix composition in a controlled setting. CDMs were generated in culture for 1–4 weeks depending on cell type, homogenized, and analyzed via label-free quantitative proteomics. No significant differences in CDM composition were found between pAFs and AcoSV40 fibroblasts, while AcoSI-1 CDMs shared 88% of proteins identified with pAFs (Fig 4A). The proteins that differed between the two samples were involved in biological processes related to metabolism and translation, based on a GO enrichment analysis. In comparison, NIH3T3s, a commonly used substitute for *Mus musculus* cells, only shared 75% of proteins with their counterpart (Fig 4B). The top 20 proteins from each cell type are listed in S1 and S2 Tables, and a full list of proteins is available in an online database [52]. We saw that a majority of the most highly expressed proteins were shared between primary *Acomys*, AcoSV40, and AcoSI-1 CDMs, but that AcoSV40 and AcoSI-1 CDMs also had high production of proteins like heat shock protein 90 and serpin H1, which promote cell proliferation and survival [53,54]. Based on these results, AcoSV40 and AcoSI-1 are representative of pAFs in experiments related to ECM deposition.

### Determining sex of immortalized *Acomys* cell lines

**Fig 5:**
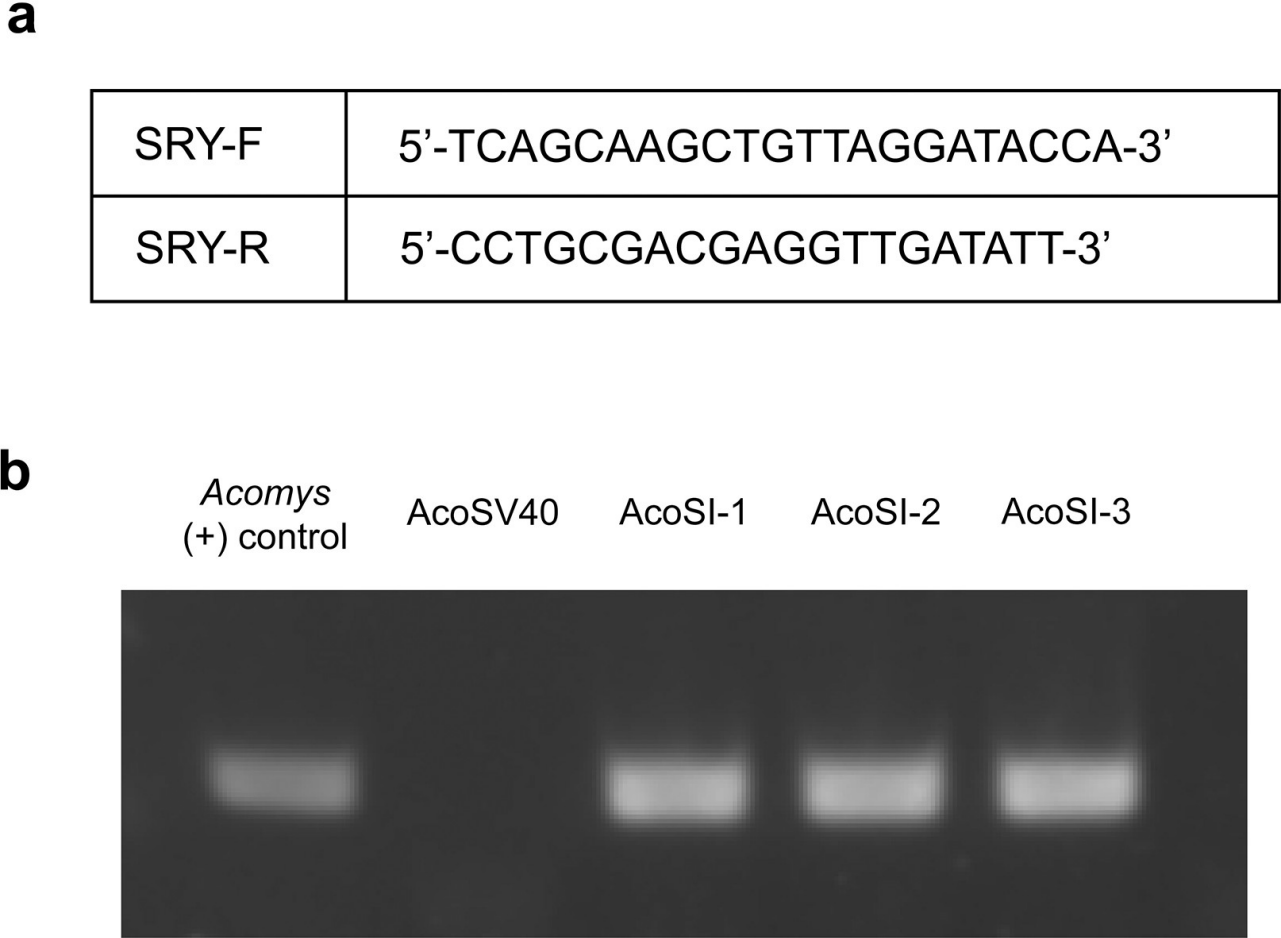
AcoSV40 cells are female while all AcoSI cells are male. (a) Forward and reverse primers specific to *Acomys* SrY. (b) PCR products for Y chromosome in all 4 cell lines compared to a DNA sample obtained from the blood of an *Acomys* adult male.

It is becoming increasingly evident that considering the sex of cell lines is important. Genes expressed on the sex chromosomes can have an impact on a cell’s biology, and cells differ according to sex, regardless of their exposure to sex hormones. The basis of differences between male and female cells and examples of known differences between male and female cells from a variety of tissues are reviewed by Shah et al [55]. Since we were unable to sex the *Acomys* pups from which the cells were isolated, we determined the sex of our immortalized lines via PCR using primers designed for the *Acomys* SRY gene. The SRY gene provides instructions for making the sex-determining region Y protein which is located on the Y chromosome and is involved in male-typical sex development. Genomic DNA was obtained from cell cultures of the four immortalized lines and the blood of a male *Acomys* and assessed for the presence of SRY (Fig 5A). DNA from AcoSV40 fibroblasts did not contain the SrY gene, while all AcoSI cell lies did, meaning AcoSV40 cells are female while all AcoSI cells are male (Fig 5B).

## Discussion

Tissue damage in humans generally results in the formation of scar tissue, which has structural and mechanical properties that differ from uninjured tissue and often impede tissue function. Regenerative organisms present an opportunity to uncover mechanisms behind scar-free healing that can improve patient outcomes following tissue damage. The spiny mouse (*Acomys*) is an exciting research organism with the most extensive regeneration capabilities of any known mammal. Unfortunately, *Acomys* research is currently limited to a handful of institutions due to the need for non-traditional animal facilities and husbandry protocols, as well as the limited access to *Acomys* vendors [1,18–20]. To increase access to *Acomys* research and reduce the use of animals in regenerative medicine research, we developed two immortalized *Acomys* fibroblast cell lines. We generated the lines through two well described methods—SV40LT transfection (“AcoSV40”) and spontaneous immortalization (“AcoSI-1”)—and assessed morphological and functional characteristics.

As mediators of matrix deposition and wound contraction, fibroblasts are likely key players in the scar-free healing of *Acomys*. *Acomys* wounds have low populations of αSMA positive myofibroblasts [2], even though there is a high concentration of TGF-β1 [3], a pro-fibrotic cytokine. *In vitro*, *Acomys* fibroblasts do not upregulate αSMA when treated with TGF-β1 [50]. These findings suggest that *Acomys* fibroblasts are protected from TGF-β1-mediated activation. Inhibition of TGF-β1 signaling *in vivo* is associated with reduced wound scarring, as demonstrated in a rabbit ear hypertrophic scarring model [56]. However, direct blocking of TGF-β through antibody-based methods have been unsuccessful due to adverse effects [57]. Uncovering mechanisms behind the response of *Acomys* fibroblasts to TGF-β1 may lead to more nuanced treatments targeting the TGF-β pathway. In addition to an altered response to TGF-β1, *Acomys* fibroblasts demonstrate an increased migration rate and decreased response to stiffness *in vitro*, which may also play roles in wound healing and fibrosis, respectively [45]. Proliferation and migration of dermal fibroblasts is a crucial step in wound healing due to the role of fibroblasts in depositing granulation tissue and beginning the proliferative phase of healing [58]. The lack of stiffness-induced myofibroblast differentiation in *Acomys* fibroblasts is a deviation from the common feed-forward mechanism between increased ECM deposition and myofibroblast activation that occurs in fibrotic disorders [59]. Understanding these intrinsic differences between *Acomys* fibroblasts and fibroblasts in non-regenerative mammals may point to new therapeutics to improve wound healing and mediate fibrosis.

AcoSV40 and AcoSI-1 immortalized *Acomys* fibroblasts present a great opportunity for researchers to uncover new mechanisms behind scar-free wound healing. The two cell lines are easier to culture than pAFs due to higher proliferation rates, consistent characteristics, and simplified media requirements (S3 Fig). Their off-the-shelf availability allows for more frequent experiments and collaboration across institutions. Although the two cell lines behave similarly, there are instances where one cell type may be preferred over the other. For example, AcoSV40 cells express puromycin N-acetyltransferase, a puromycin resistance gene commonly used in selection of transformed mammalian cells. This prevents the use of puromycin selection in any further genetic modifications of these cells, making the AcoSI-1 cells preferable if using lentiviral vectors with puromycin resistance. In addition, AcoSI-1 cells may be a more appropriate comparison when compared to NIH3T3 fibroblasts because they are both spontaneously immortalized lines and have low αSMA expression in standard culture conditions. However, if αSMA expression is desired under standard culture conditions, AcoSV40 fibroblasts would be preferred over AcoSI-1. Like αSMA, other proteins may be differentially expressed between the two cell lines and a decision between the two lines will need to be made based on the proteins of interest for specific experiments. Finally, AcoSV40 cells are female and AcoSI-1 cells are male, which may affect cell line choice depending on research questions, as there are differences between male and female cells from a variety of tissues [55].

To increase access to *Acomys* research and reduce the use of animals in regenerative medicine research, we developed two immortalized *Acomys* fibroblast cell lines and confirmed that morphological and functional characteristics were representative of pAFs. We believe the availability of these cells will contribute to the field’s understanding of mammalian regeneration.

## Supporting information

S1 Fig Confirmation of expression of SV40 and puromycin resistance gene. (a) Primer sequences used to analyze transgene expression, provided by ALSTEM, Inc. (b) PCR products to confirm transgene expression in AcoSV40 cells. Lane 1. MEF Sample for SV40; Lanes 2 and 5, ladder; Lane 3 and 6, positive control; Lane 4, MEF Sample for puromycin. After amplifying with primers SV40-F/R and puro-F/R, respectively, the MEF cells showed 112 bp bands for SV40 and 198 bp bands for puromycin resistance gene.
(TIF)

S2 Fig AcoSV40 and AcoSI-1 fibroblasts maintain constant proliferation rates when cultured in a standard media formulation (DMEM, 10%FBS, 1%PenStrep). Fibroblasts were seeded at a constant cell density (5,300 cells/cm^2^) and passaged every 3 days.
(TIF)

S3 Fig Primary *Acomys* fibroblasts demonstrate faster proliferation compared to primary *Mus* fibroblasts. Growth curves for early passage primary *Acomys* (blue) and *Mus musculus* (orange) fibroblasts. Fibroblasts were seeded at a constant cell density (5,300 cells/cm^2^) and passaged at 80–90% confluency.
(TIF)

S4 Fig AcoSV40 and AcoSI-1 fibroblasts do not notably upregulate αSMA in response to TGF-β treatment. (a) Western blot showing αSMA and GAPDH expression in AcoSV40, AcoSI-1, and NIH3T3 fibroblasts in standard media, serum free media, or serum free media + 2ng/mL TGF-β. (b) Fold change of αSMA production with TGF-β treatment in serum free media compared to serum free media alone. Fibroblasts were serum starved for 12 hours, then treated with serum free media +/- 2ng/mL TGF-β for 48 hours. Band intensities for αSMA were quantified in ImageJ and normalized to GAPDH. Then, the relative αSMA band intensities for the TGF-β treatment groups were compared to serum free media by calculating fold change.
(TIF)

S1 Table 20 most highly expressed proteins in CDM samples from primary *Acomys*, AcoSV40, and AcoSI-1 fibroblasts. Proteins were ordered based on sumPEP score output from Proteome Discoverer, where a higher sumPEP score indicates higher abundance.
(DOCX)

S2 Table 20 most highly expressed proteins in CDM samples from primary *Mus* and NIH3T3 fibroblasts. Proteins were ordered based on sumPEP score output from Proteome Discoverer, where a higher sumPEP score indicates higher abundance.
(DOCX)

## References

[1] Gaire J, Varholick JA, Rana S, Sunshine MD, Doré S, Barbazuk WB, et al. Spiny mouse (Acomys): an emerging research organism for regenerative medicine with applications beyond the skin. npj Regenerative Medicine 2021 6:1 [Internet]. 2021 Jan 4 [cited 2021 Jul 20];6(1):1–6. Available from: https://www.nature.com/articles/s41536-020-00111-1. doi: 10.1038/s41536-020-00111-1 33397999 PMC7782534

[2] Seifert AW, Kiama SG, Seifert MG, Goheen JR, Palmer TM, Maden M. Skin shedding and tissue regeneration in African spiny mice (Acomys). Nature 2012 489:7417 [Internet]. 2012 Sep 26 [cited 2022 Jan 31];489(7417):561–5. Available from: https://www.nature.com/articles/nature11499. doi: 10.1038/nature11499 23018966 PMC3480082

[3] Brant JO, Lopez MC, Baker H v., Barbazuk WB, Maden M. A Comparative Analysis of Gene Expression Profiles during Skin Regeneration in Mus and Acomys. PLoS One [Internet]. 2015 Nov 1 [cited 2022 Jan 31];10(11):e0142931. Available from: https://journals.plos.org/plosone/article?id=10.1371/journal.pone.0142931. doi: 10.1371/journal.pone.0142931 26606282 PMC4659537

[4] Brant JO, Yoon JH, Polvadore T, Barbazuk WB, Maden M. Cellular events during scar-free skin regeneration in the spiny mouse, Acomys. Wound Repair and Regeneration [Internet]. 2016 Jan 1 [cited 2022 Jan 31];24(1):75–88. Available from: https://onlinelibrary.wiley.com/doi/full/10.1111/wrr.12385. 26606280 10.1111/wrr.12385

[5] Maden M. Optimal skin regeneration after full thickness thermal burn injury in the spiny mouse, Acomys cahirinus. Burns. 2018 Sep 1;44(6):1509–20. doi: 10.1016/j.burns.2018.05.018 29903601

[6] Maden M, Brant JO. Insights into the regeneration of skin from Acomys, the spiny mouse. Exp Dermatol [Internet]. 2019 Apr 1 [cited 2022 Jan 31];28(4):436–41. Available from: https://onlinelibrary.wiley.com/doi/full/10.1111/exd.13847. 30457673 10.1111/exd.13847

[7] Yoon JH, Cho K, Garrett TJ, Finch P, Maden M. Comparative Proteomic Analysis in Scar-Free Skin Regeneration in Acomys cahirinus and Scarring Mus musculus. Scientific Reports 2020 10:1 [Internet]. 2020 Jan 13 [cited 2022 Jan 31];10(1):1–16. Available from: https://www.nature.com/articles/s41598-019-56823-y. 31932597 10.1038/s41598-019-56823-y PMC6957500

[8] Matias Santos D, Rita AM, Casanellas I, Brito Ova A, Araújo IM, Power D, et al. Ear wound regeneration in the African spiny mouse *Acomys cahirinus*. Regeneration [Internet]. 2016 Feb 1 [cited 2019 Jan 16];3(1):52–61. Available from: http://doi.wiley.com/ doi: 10.1002/reg2.50 27499879 PMC4857749

[9] Gawriluk TR, Simkin J, Thompson KL, Biswas SK, Clare-Salzler Z, Kimani JM, et al. Comparative analysis of ear-hole closure identifies epimorphic regeneration as a discrete trait in mammals. Nat Commun [Internet]. 2016 Apr 25 [cited 2020 Nov 11];7(1):1–16. Available from: www.nature.com/naturecommunications. doi: 10.1038/ncomms11164 27109826 PMC4848467

[10] Simkin J, Gawriluk TR, Gensel JC, Seifert AW. Macrophages are necessary for epimorphic regeneration in African spiny mice. Elife. 2017 May 16;6. doi: 10.7554/eLife.24623 28508748 PMC5433844

[11] Maden M, Brant JO, Rubiano A, Sandoval AGW, Simmons C, Mitchell R, et al. Perfect chronic skeletal muscle regeneration in adult spiny mice, Acomys cahirinus. Scientific Reports 2018 8:1 [Internet]. 2018 Jun 11 [cited 2022 Jan 31];8(1):1–14. Available from: https://www.nature.com/articles/s41598-018-27178-7. doi: 10.1038/s41598-018-27178-7 29892004 PMC5995887

[12] Okamura DM, Brewer CM, Wakenight P, Bahrami N, Bernardi K, Tran A, et al. Spiny mice activate unique transcriptional programs after severe kidney injury regenerating organ function without fibrosis. iScience. 2021 Nov 19;24(11):103269. doi: 10.1016/j.isci.2021.103269 34849462 PMC8609232

[13] Qi Y, Dasa O, Maden M, Vohra R, Batra A, Walter G, et al. Functional heart recovery in an adult mammal, the spiny mouse. Int J Cardiol. 2021 Sep 1;338:196–203. doi: 10.1016/j.ijcard.2021.06.015 34126132

[14] Peng H, Shindo K, Donahue RR, Gao E, Ahern BM, Levitan BM, et al. Adult spiny mice (Acomys) exhibit endogenous cardiac recovery in response to myocardial infarction. npj Regenerative Medicine 2021 6:1 [Internet]. 2021 Nov 17 [cited 2022 Jan 31];6(1):1–15. Available from: https://www.nature.com/articles/s41536-021-00186-4. doi: 10.1038/s41536-021-00186-4 34789749 PMC8599698

[15] Koopmans T, van Beijnum H, Roovers EF, Tomasso A, Malhotra D, Boeter J, et al. Ischemic tolerance and cardiac repair in the spiny mouse (Acomys). npj Regenerative Medicine 2021 6:1 [Internet]. 2021 Nov 17 [cited 2022 Jan 31];6(1):1–16. Available from: https://www.nature.com/articles/s41536-021-00188-2. doi: 10.1038/s41536-021-00188-2 34789755 PMC8599451

[16] Streeter KA, Sunshine MD, Brant JO, Sandoval AGW, Maden M, Fuller DD. Molecular and histologic outcomes following spinal cord injury in spiny mice, Acomys cahirinus. Journal of Comparative Neurology [Internet]. 2020 Jun 15 [cited 2020 Aug 12];528(9):1535–47. Available from: https://pubmed.ncbi.nlm.nih.gov/31820438/. doi: 10.1002/cne.24836 31820438 PMC7523565

[17] Nogueira-Rodrigues J, Leite SC, Pinto-Costa R, Sousa SC, Luz LL, Sintra MA, et al. Rewired glycosylation activity promotes scarless regeneration and functional recovery in spiny mice after complete spinal cord transection. Dev Cell. 2022 Jan 4. doi: 10.1016/j.devcel.2021.12.008 34986324

[18] Pinheiro G, Prata DF, Araújo IM, Tiscornia G. The African spiny mouse (Acomys spp.) as an emerging model for development and regeneration. Lab Anim. 2018 Dec 1;52(6):565–76. doi: 10.1177/0023677218769921 29699452

[19] Haughton CL, Gawriluk TR, Seifert AW. The Biology and Husbandry of the African Spiny Mouse (Acomys cahirinus)and the Research Uses of a Laboratory Colony. J Am Assoc Lab Anim Sci [Internet]. 2016 Jan 1 [cited 2022 Feb 1];55(1):9. Available from: /pmc/articles/PMC4747004/. 26817973 PMC4747004

[20] Dickinson H, Walker D. Managing a colony of spiny mice (. Australian and New Zealand Council for the Care of Animals in Research and Training (ANZCCART) News. 2007 Jan 1;20:4–11.

[21] Hayflick L, Moorhead PS. The serial cultivation of human diploid cell strains. Exp Cell Res. 1961 Dec 1;25(3):585–621. doi: 10.1016/0014-4827(61)90192-6 13905658

[22] Goldstein S. Replicative Senescence: the Human Fibroblast Comes of Age. Science (1979) [Internet]. 1990 [cited 2022 Feb 13];249(4973):1129–33. Available from: https://www.science.org/doi/abs/10.1126/science.220411 2204114 10.1126/science.2204114

[23] Maden M, Varholick JA. Model systems for regeneration: the spiny mouse, Acomys cahirinus. Development [Internet]. 2020 [cited 2022 Feb 14];147(4). Available from: /pmc/articles/PMC7055364/. doi: 10.1242/dev.167718 32098790 PMC7055364

[24] Pennello A, Taylor J, Matlack R, Karp J, Riggs J. Spiny mice (Acomys cahirinus) do not respond to thymus-independent type 2 antigens. Dev Comp Immunol. 2006 Jan 1;30(12):1181–90. doi: 10.1016/j.dci.2006.03.007 16698082

[25] Ozer HL, Banga SS, Dasgupta T, Houghton J, Hubbard K, Jha KK, et al. SV40-mediated immortalization of human fibroblasts. Exp Gerontol. 1996 Jan 1;31(1–2):303–10. doi: 10.1016/0531-5565(95)00024-0 8706800

[26] Nobre A, Kalve I, Cesnulevicius K, Rangancokova D, Ratzka A, Halfer N, et al. Characterization and differentiation potential of rat ventral mesencephalic neuronal progenitor cells immortalized with SV40 large T antigen. Cell Tissue Res [Internet]. 2010 Feb 23 [cited 2022 Feb 14];340(1):29–43. Available from: https://link.springer.com/article/10.1007/s00441-010-0933-4. doi: 10.1007/s00441-010-0933-4 20177706

[27] Chen Y, Hu S, Wang M, Zhao B, Yang N, Li J, et al. Characterization and Establishment of an Immortalized Rabbit Melanocyte Cell Line Using the SV40 Large T Antigen. International Journal of Molecular Sciences 2019, Vol 20, Page 4874 [Internet]. 2019 Sep 30 [cited 2022 Feb 14];20(19):4874. Available from: https://www.mdpi.com/1422-0067/20/19/4874/htm. doi: 10.3390/ijms20194874 31575080 PMC6802187

[28] Whitehead RH, Robinson PS. Establishment of conditionally immortalized epithelial cell lines from the intestinal tissue of adult normal and transgenic mice. Am J Physiol Gastrointest Liver Physiol [Internet]. 2009 Mar [cited 2022 Feb 14];296(3):455–60. Available from: https://journals.physiology.org/doi/abs/ doi: 10.1152/ajpgi.90381.2008 19109407 PMC2660172

[29] Hara E, Tsurui H, Shinozaki A, Nakada S, Oda K. Cooperative effect of antisense-Rb and antisense-p53 oligomers on the extension of life span in human diploid fibroblasts, TIG-1. Biochem Biophys Res Commun. 1991 Aug 30;179(1):528–34. doi: 10.1016/0006-291x(91)91403-y 1909121

[30] Counter CM, Avilion AA, Lefeuvre CE, Stewart NG, Greider CW, Harley CB, et al. Telomere shortening associated with chromosome instability is arrested in immortal cells which express telomerase activity. EMBO J [Internet]. 1992 [cited 2022 Feb 13];11(5):1921. Available from: /pmc/articles/PMC556651/?report=abstract. doi: 10.1002/j.1460-2075.1992.tb05245.x 1582420 PMC556651

[31] Ozer HL, Slater ML, Dermody JJ, Mandei4 M. Replication of simian virus 40 DNA in normal human fibroblasts and in fibroblasts from xeroderma pigmentosum. J Virol [Internet]. 1981 Aug [cited 2022 Feb 13];39(2):481–9. Available from: https://journals.asm.org/doi/abs/10.1128/jvi.39.2.481-489.1981. 6268837 10.1128/jvi.39.2.481-489.1981 PMC171358

[32] Small MB, Gluzmant Y, Ozer HL. Enhanced transformation of human fibroblasts by origin-defective simian virus 40. Nature. 1982;296. doi: 10.1038/296671a0 6280060

[33] Katakura Y, Alam S, Shirahata S. Immortalization by gene transfection. Methods Cell Biol. 1998;57:69–91. doi: 10.1016/s0091-679x(08)61573-3 9648100

[34] Justin McCormick J, Maher VM. Towards an understanding of the malignant transformation of diploid human fibroblasts. Mutat Res [Internet]. 1988 [cited 2022 Feb 14];199(2):273–91. Available from: https://pubmed.ncbi.nlm.nih.gov/3287148/. doi: 10.1016/0027-5107(88)90209-6 3287148

[35] TODARO GJ GREEN H. Quantitative studies of the growth of mouse embryo cells in culture and their development into established lines. J Cell Biol [Internet]. 1963 [cited 2020 Nov 18];17(2):299–313. Available from: /pmc/articles/PMC2106200/?report=abstract. 13985244 10.1083/jcb.17.2.299 PMC2106200

[36] Fridman AL, Tainsky MA. Critical pathways in cellular senescence and immortalization revealed by gene expression profiling. Oncogene [Internet]. 2008 Oct 9 [cited 2022 Mar 9];27(46):5975–87. Available from: https://pubmed.ncbi.nlm.nih.gov/18711403/. doi: 10.1038/onc.2008.213 18711403 PMC3843241

[37] Harvey M, Sands AT, Weiss RS, Hegi ME, Wiseman RW, Pantazis P, et al. In vitro growth characteristics of embryo fibroblasts isolated from p53-deficient mice. Oncogene. 1993 Sep;8(9):2457–67. 8103211

[38] Harvey DM, Levine AJ. p53 alteration is a common event in the spontaneous immortalization of primary BALB/c murine embryo fibroblasts. Genes Dev [Internet]. 1991 [cited 2022 Mar 9];5(12B):2375–85. Available from: https://pubmed.ncbi.nlm.nih.gov/1752433/. doi: 10.1101/gad.5.12b.2375 1752433

[39] Zhang H, Pan KH, Cohen SN. Senescence-specific gene expression fingerprints reveal cell-type-dependent physical clustering of up-regulated chromosomal loci. Proc Natl Acad Sci U S A [Internet]. 2003 Mar 18 [cited 2022 Mar 9];100(6):3251–6. Available from: www.pnas.orgcgidoi10.1073pnas.2627983100. 12626749 10.1073/pnas.2627983100 PMC152278

[40] Zhang H, Herbert BS, Pan KH, Shay JW, Cohen SN. Disparate effects of telomere attrition on gene expression during replicative senescence of human mammary epithelial cells cultured under different conditions. Oncogene 2004 23:37 [Internet]. 2004 Jun 14 [cited 2022 Mar 9];23(37):6193–8. Available from: https://www.nature.com/articles/1207834. doi: 10.1038/sj.onc.1207834 15195144

[41] Zhao X, Zhao Q, Luo Z, Yu Y, Xiao NA, Sun X, et al. Spontaneous immortalization of mouse liver sinusoidal endothelial cells. Int J Mol Med [Internet]. 2015 Mar 1 [cited 2022 Mar 9];35(3):617–24. Available from: http://www.spandidos-publications.com/10.3892/ijmm.2015.2067/abstract. doi: 10.3892/ijmm.2015.2067 25585915 PMC4314414

[42] Diebold Y, Calonge M, de Salamanca AE, Callejo S, Corrales RM, Sáez V, et al. Characterization of a Spontaneously Immortalized Cell Line (IOBA-NHC) from Normal Human Conjunctiva. Invest Ophthalmol Vis Sci. 2003 Oct 1;44(10):4263–74. doi: 10.1167/iovs.03-0560 14507870

[43] Gillio-Meina C, Swan CL, Crellin NK, Stocco DM, Chedrese PJ. Generation of Stable Cell Lines by Spontaneous Immortalization of Primary Cultures of Porcine Granulosa Cells. Mol Reprod Dev. 2000;57:366–74. doi: 10.1002/1098-2795(200012)57:4&lt;366::AID-MRD9&gt;3.0.CO;2-B 11066066

[44] Christman SA, Kong BW, Landry MM, Kim H, Foster DN. Contributions of differential p53 expression in the spontaneous immortalization of a chicken embryo fibroblast cell line. BMC Cell Biol [Internet]. 2006 Jun 30 [cited 2022 Mar 9];7(1):1–12. Available from: https://bmcmolcellbiol.biomedcentral.com/articles/10.1186/1471-2121-7-27 16813656 10.1186/1471-2121-7-27 PMC1533818

[45] Stewart DC, Serrano PN, Rubiano A, Yokosawa R, Sandler J, Mukhtar M, et al. Unique behavior of dermal cells from regenerative mammal, the African Spiny Mouse, in response to substrate stiffness. J Biomech [Internet]. 2018 Nov 16 [cited 2019 Jan 1];81:149–54. Available from: https://www.sciencedirect.com/science/article/pii/S002192901830767X. doi: 10.1016/j.jbiomech.2018.10.005 30361050

[46] Butler JP, Toli-Nørrelykke IM, Fabry B, Fredberg JJ. Traction fields, moments, and strain energy that cells exert on their surroundings. Am J Physiol Cell Physiol [Internet]. 2002 [cited 2022 Sep 13];282(3). Available from: https://pubmed.ncbi.nlm.nih.gov/11832345/. doi: 10.1152/ajpcell.00270.2001 11832345

[47] Negmadjanov U, Godic Z, Rizvi F, Emelyanova L, Ross G, Richards J, et al. TGF-β1-Mediated Differentiation of Fibroblasts Is Associated with Increased Mitochondrial Content and Cellular Respiration. PLoS One [Internet]. 2015 Apr 7 [cited 2022 Mar 23];10(4). Available from: /pmc/articles/PMC4388650/. 10.1371/journal.pone.0123046 PMC4388650 25849590

[48] Abdalla M, Thompson LA, Gurley E, Burke S, Ujjin J, Newsome R, et al. Dasatinib inhibits TGFβ-induced myofibroblast differentiation through Src-SRF Pathway. Eur J Pharmacol. 2015 Dec 15;769:134–42. 26548624 10.1016/j.ejphar.2015.11.008 PMC4679708

[49] Cheng W, Xu R, Li D, Bortolini C, He J, Dong M, et al. Artificial extracellular matrix delivers TGFb1 regulating myofibroblast differentiation. RSC Adv [Internet]. 2016 Feb 23 [cited 2022 Mar 23];6(26):21922–8. Available from: https://pubs.rsc.org/en/content/articlehtml/2016/ra/c5ra26164c.

[50] Brewer CM, Nelson BR, Wakenight P, Collins SJ, Okamura DM, Dong XR, et al. Adaptations in Hippo-Yap signaling and myofibroblast fate underlie scar-free ear appendage wound healing in spiny mice. Dev Cell [Internet]. 2021 Oct [cited 2021 Nov 21];56(19):2722–2740.e6. Available from: https://pubmed.ncbi.nlm.nih.gov/34610329/. doi: 10.1016/j.devcel.2021.09.008 34610329 PMC8623355

[51] Naba A, Clauser KR, Ding H, Whittaker CA, Carr SA, Hynes RO. The Extracellular Matrix: Tools and Insights for the “Omics” Era. Matrix Biol [Internet]. 2016 Jan 1 [cited 2022 May 12];49:10. Available from: /pmc/articles/PMC5013529/. doi: 10.1016/j.matbio.2015.06.003 26163349 PMC5013529

[52] Dill M, Kamat M, Basso K, Simmons C. Composition of Cell-Derived Matrices from Acomys cahirinus and Mus musculus—Label Free Quantitative Proteomics. 2022;1.

[53] Ren X, Li T, Zhang W, Yang X. Targeting Heat-Shock Protein 90 in Cancer: An Update on Combination Therapy. Cells [Internet]. 2022 Aug 1 [cited 2023 Jun 10];11(16). Available from: /pmc/articles/PMC9406578/. doi: 10.3390/cells11162556 36010632 PMC9406578

[54] Tian S, Peng P, Li J, Deng H, Zhan N, Zeng Z, et al. SERPINH1 regulates EMT and gastric cancer metastasis via the Wnt/β-catenin signaling pathway. Aging (Albany NY) [Internet]. 2020 Feb 2 [cited 2023 Jun 10];12(4):3574. Available from: /pmc/articles/PMC7066881/. 32091407 10.18632/aging.102831 PMC7066881

[55] Shah K, McCormack CE, Bradbury NA. Do you know the sex of your cells? Am J Physiol Cell Physiol [Internet]. 2014 Jan 1 [cited 2022 May 3];306(1):C3. Available from: /pmc/articles/PMC3919971/. doi: 10.1152/ajpcell.00281.2013 24196532 PMC3919971

[56] Wang YW, Liou NH, Cherng JH, Chang SJ, Ma KH, Fu E, et al. siRNA-targeting transforming growth factor-β type I receptor reduces wound scarring and extracellular matrix deposition of scar tissue. J Invest Dermatol [Internet]. 2014 [cited 2022 Sep 13];134(7):2016–25. Available from: https://pubmed.ncbi.nlm.nih.gov/24670383/. 24670383 10.1038/jid.2014.84

[57] Henderson NC, Rieder F, Wynn TA. Fibrosis: from mechanisms to medicines. Nature 2020 587:7835 [Internet]. 2020 Nov 25 [cited 2022 Sep 14];587(7835):555–66. Available from: https://www.nature.com/articles/s41586-020-2938-9. doi: 10.1038/s41586-020-2938-9 33239795 PMC8034822

[58] Landén NX, Li D, Ståhle M. Transition from inflammation to proliferation: a critical step during wound healing. Cellular and Molecular Life Sciences [Internet]. 2016 Oct 1 [cited 2022 Sep 14];73(20):3861. Available from: /pmc/articles/PMC5021733/. doi: 10.1007/s00018-016-2268-0 27180275 PMC5021733

[59] Liu F, Mih JD, Shea BS, Kho AT, Sharif AS, Tager AM, et al. Feedback amplification of fibrosis through matrix stiffening and COX-2 suppression. J Cell Biol [Internet]. 2010 Aug 8 [cited 2022 Dec 13];190(4):693. Available from: /pmc/articles/PMC2928007/. doi: 10.1083/jcb.201004082 20733059 PMC2928007

