# Supplementary figures and images for: Generation and characterization of two immortalized dermal fibroblast cell lines from the spiny mouse (Acomys)
Source: PLoS One. 2023 Jul 7;18(7):e0280169. doi: 10.1371/journal.pone.0280169 (PMC10328323; doi:10.1371/journal.pone.0280169)

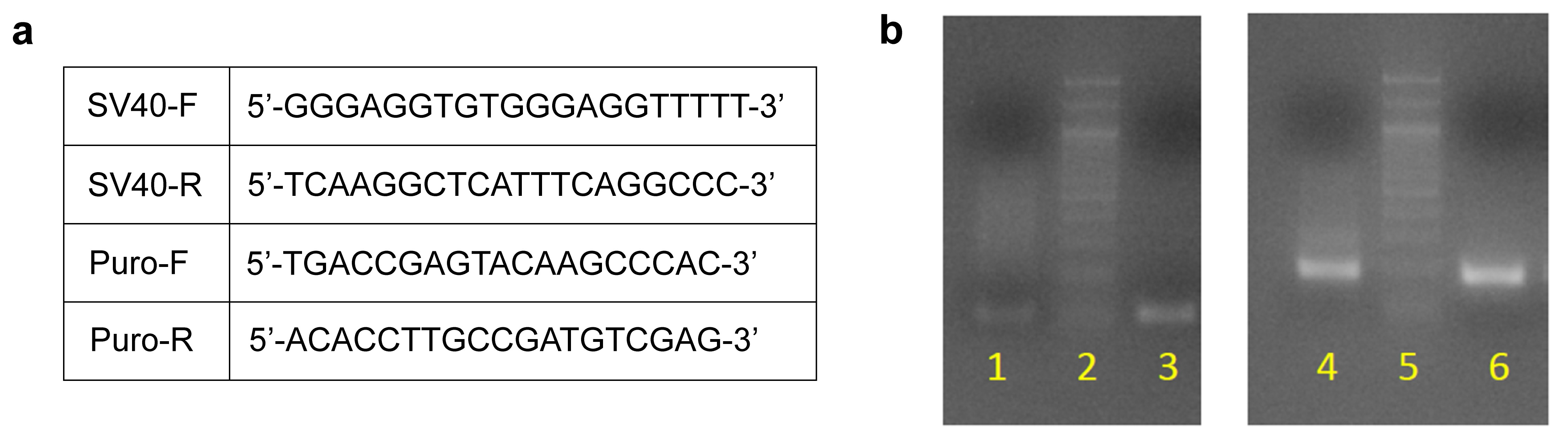

Supplement: S1 Fig — (a) Primer sequences used to analyze transgene expression, provided by ALSTEM, Inc. (b) PCR products to confirm transgene expression in AcoSV40 cells. Lane 1. MEF Sample for SV40; Lanes 2 and 5, ladder; Lane 3 and 6, positive control; Lane 4, MEF Sample for puromycin. After amplifying with primers SV40-F/R and puro-F/R, respectively, the MEF cells showed 112 bp bands for SV40 and 198 bp bands for puromycin resistance gene. (TIF) [file pone.0280169.s001.tif]

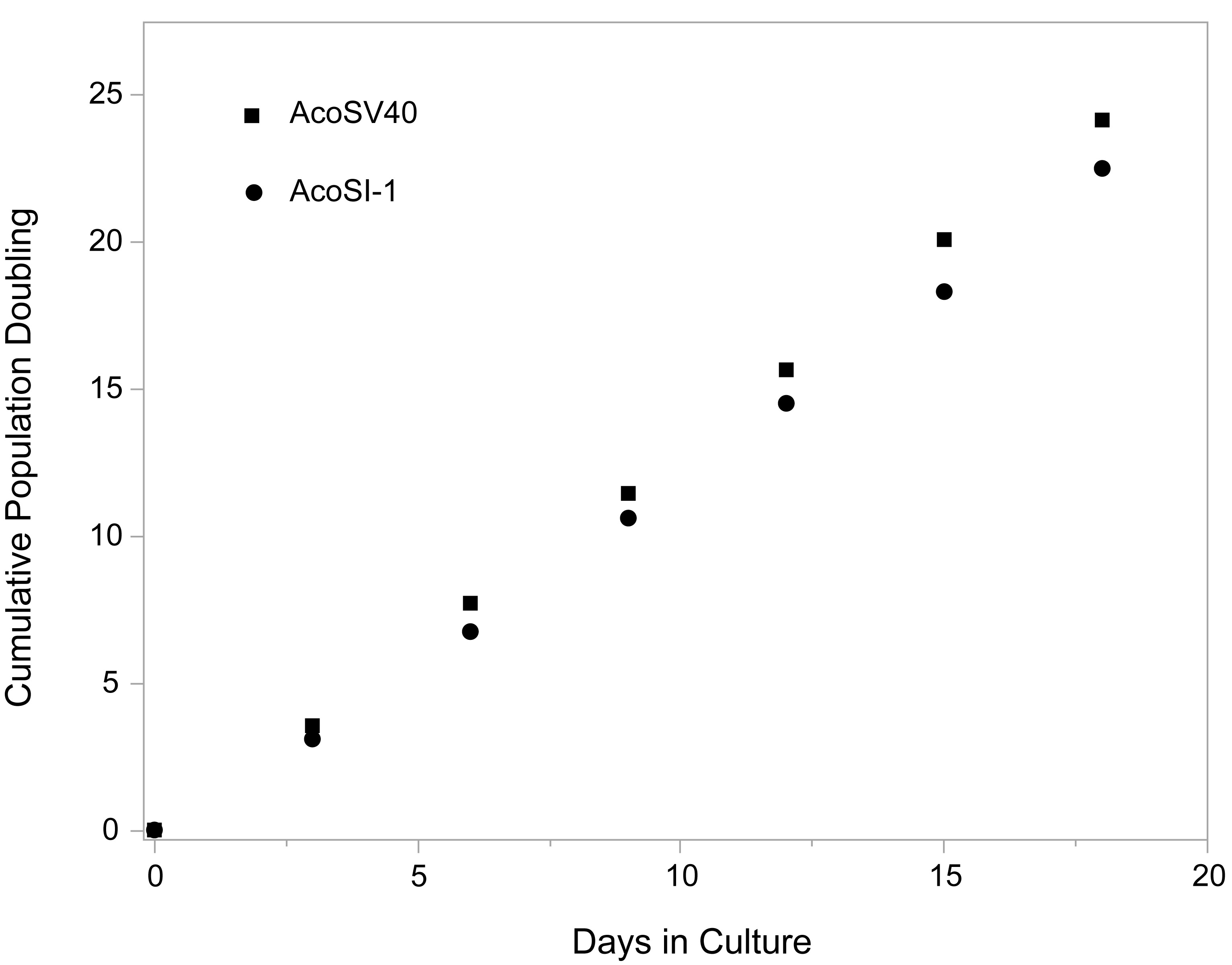

Supplement: S2 Fig — Fibroblasts were seeded at a constant cell density (5,300 cells/cm2) and passaged every 3 days. (TIF) [file pone.0280169.s002.tif]

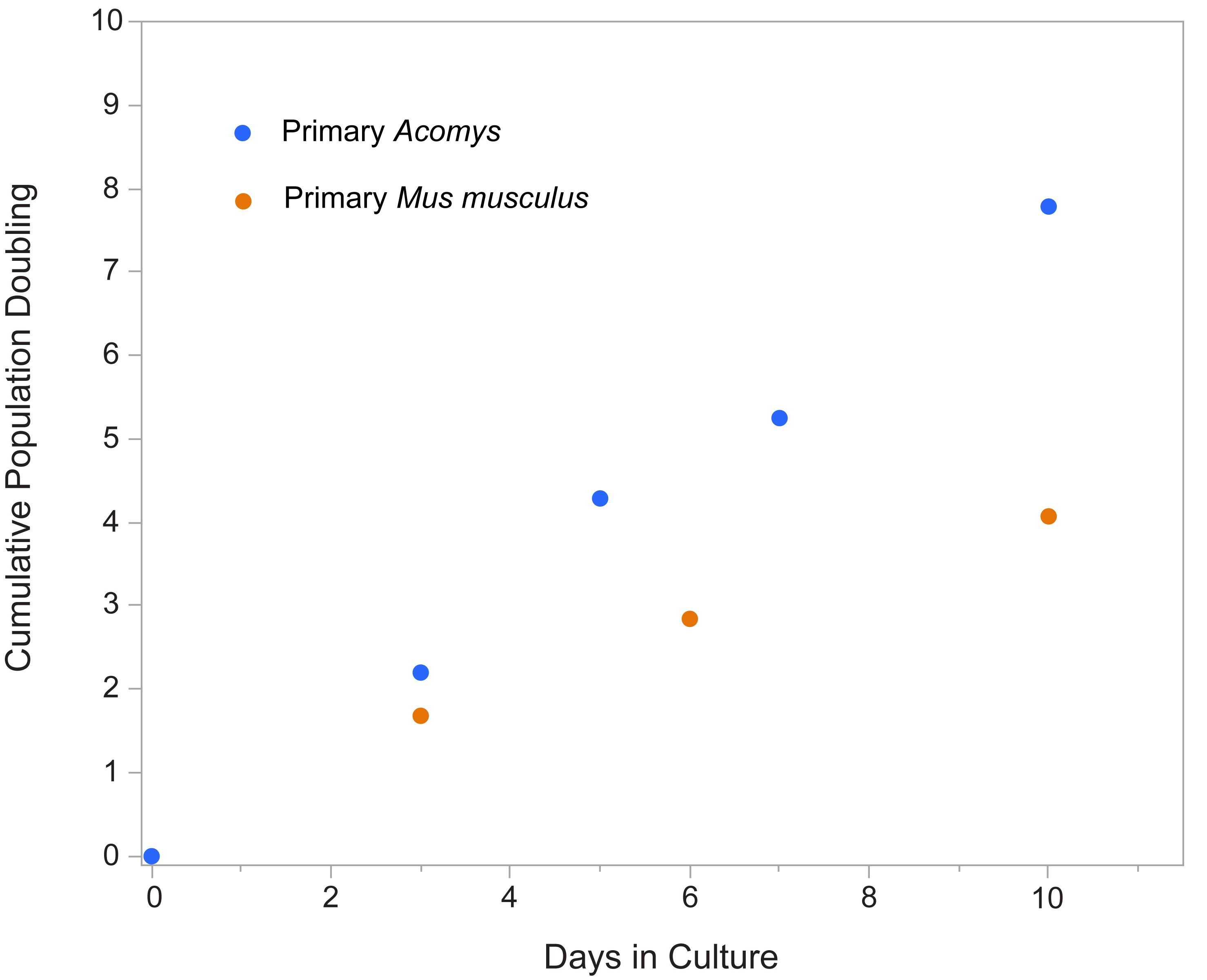

Supplement: S3 Fig — Growth curves for early passage primary Acomys (blue) and Mus musculus (orange) fibroblasts. Fibroblasts were seeded at a constant cell density (5,300 cells/cm2) and passaged at 80–90% confluency. (TIF) [file pone.0280169.s003.tif]

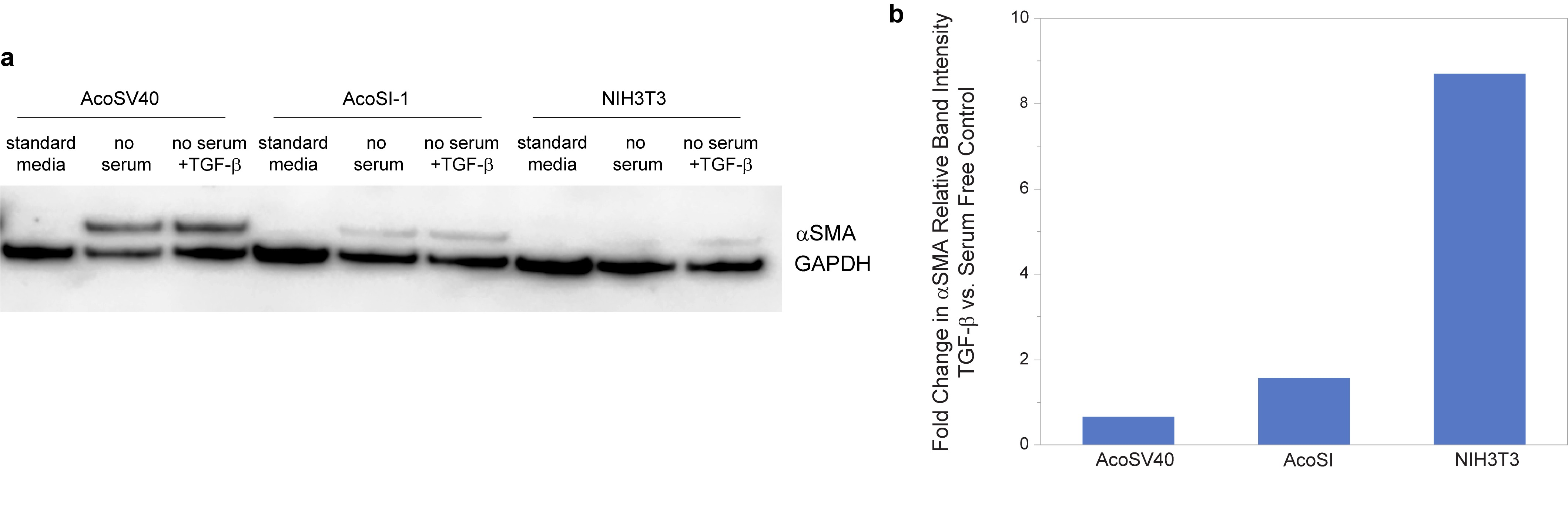

Supplement: S4 Fig — (a) Western blot showing αSMA and GAPDH expression in AcoSV40, AcoSI-1, and NIH3T3 fibroblasts in standard media, serum free media, or serum free media + 2ng/mL TGF-β. (b) Fold change of αSMA production with TGF-β treatment in serum free media compared to serum free media alone. Fibroblasts were serum starved for 12 hours, then treated with serum free media +/- 2ng/mL TGF-β for 48 hours. Band intensities for αSMA were quantified in ImageJ and normalized to GAPDH. Then, the relative αSMA band intensities for the TGF-β treatment groups were compared to serum free media by calculating fold change. (TIF) [file pone.0280169.s004.tif]
