# Supplementary material for: Generation and characterization of two immortalized dermal fibroblast cell lines from the spiny mouse (Acomys)
Source: PLoS One. 2023 Jul 7;18(7):e0280169. doi: 10.1371/journal.pone.0280169 (PMC10328323; doi:10.1371/journal.pone.0280169)
Supplement: S1 Table — Proteins were ordered based on sumPEP score output from Proteome Discoverer, where a higher sumPEP score indicates higher abundance. (DOCX) [file pone.0280169.s005.docx]

| **Primary *Acomys*** | **AcoSV40** | **AcoSI-1** |
| --- | --- | --- |
| Fibronectin | Fibronectin | Fibronectin |
| Myosin-9 | Vimentin | Myosin-9 |
| Basement membrane-specific heparan sulfate proteoglycan core protein | Basement membrane-specific heparan sulfate proteoglycan core protein | Collagen alpha-1(XII) chain |
| Collagen alpha-1(XII) chain | Tubulin beta-5 chain | Vimentin |
| Fibrillin-1 | Tubulin beta-4B chain | Basement membrane-specific heparan sulfate proteoglycan core protein |
| Vimentin | Actin, cytoplasmic 1 | Pyruvate Kinase PKM |
| Periostin | Tubulin alpha-1B chain | Filamin-A |
| Myosin-10 | Tubulin beta-4A chain | Actin, cytoplasmic 2 |
| Actin, cytoplasmic 2 | Tubulin alpha-1C chain | Actin, cytoplasmic 1 |
| Actin, cytoplasmic 1 | Actin, cytoplasmic 2 | Tubulin beta-5 chain |
| Albumin | Tubulin alpha-1D chain | Heat shock protein HSP 90-beta |
| Filamin A | Albumin | Clathrin heavy chain 1 |
| Tubulin beta-5 chain | Tubulin beta-6 chain | Tubulin alpha-1B chain |
| Tenascin | Pyruvate kinase PKM | Tubulin beta-4B chain |
| Pyruvate kinase PKM | Heat shock protein HSP 90-beta | Tubulin alpha-1A chain |
| Tubulin beta-2A chain | Myosin-9 | Tubulin alpha-1C chain |
| Thrombospondin-1 | ATP synthase subunit beta, mitochondrial | Periostin |
| Periostin | Histone H4 | Tubulin beta-6 chain |
| Tubulin beta-4A chain | Collagen alpha1(I) chain | Albumin |
| Tubulin alpha-1D chain | Serpin H1 | Serpin H1 |
