# Supplementary material for: Generation and characterization of two immortalized dermal fibroblast cell lines from the spiny mouse (Acomys)
Source: PLoS One. 2023 Jul 7;18(7):e0280169. doi: 10.1371/journal.pone.0280169 (PMC10328323; doi:10.1371/journal.pone.0280169)
Supplement: S2 Table — Proteins were ordered based on sumPEP score output from Proteome Discoverer, where a higher sumPEP score indicates higher abundance. (DOCX) [file pone.0280169.s006.docx]

| **Primary *Mus*** | **NIH3T3** |
| --- | --- |
| Fibronectin | Fibronectin |
| Myosin-9 | Myosin-9 |
| Basement membrane-specific heparan sulfate proteoglycan core protein | Vimentin |
| Collagen alpha-1(XII) chain | Pyruvate kinase PKM |
| Fibrillin-1 | Tubulin beta-5 chain |
| Tenascin | Basement membrane-specific heparan sulfate proteoglycan core protein |
| Vimentin | Heat shock protein HSP 90-beta |
| Periostin | Collagen alpha-1(XII) chain |
| Albumin | Tubulin alpha-1B chain |
| Actin, cytoplasmic 2 | Tubulin alpha-1D chain |
| Actin, cytoplasmic 1 | Tubulin beta-4B chain |
| Fibulin-2 | Tubulin alpha-1c chain |
| Filamin-1 | Actin, cytoplasmic 2 |
| Tubulin beta-5 chain | Actin, cytoplasmic 1 |
| Cytoskeleton-associated protein 4 | Albumin |
| Pyruvate kinase PKM | Clathrin heavy chain 1 |
| Plectin | Tubulin beta-3 chain |
| Tubulin beta-4B | Tubulin beta-6 chain |
| Voltage-dependent anion-selective channel protein 1 | Serpin H1 |
| Actin, gamma-enteric smooth muscle | Transitional endoplasmic reticulum ATPase |
